# Active Iron‐Drug Nanocomplexes Improve Photodynamic and Photothermal Cancer Therapy by Mitigating Tumor Hypoxia and Counteracting Tumor Heat Resistance

**DOI:** 10.1002/adhm.202404485

**Published:** 2025-02-23

**Authors:** Yuying Yin, Ka Hong Wong, Liewei Wen, Meiwan Chen

**Affiliations:** ^1^ State Key Laboratory of Quality Research in Chinese Medicine Institute of Chinese Medical Sciences University of Macau Macau SAR 999078 China; ^2^ MoE Frontiers Science Center for Precision Oncology University of Macau Macau SAR 999078 China; ^3^ Guangdong Provincial Key Laboratory of Tumor Interventional Diagnosis and Treatment Zhuhai People's Hospital (Zhuhai Clinical Medical College of Jinan University) Jinan University Zhuhai Guangdong 519000 China

**Keywords:** apigenin, hypericin, photodynamic therapy, photothermal therapy

## Abstract

Photodynamic therapy (PDT) and photothermal therapy (PTT) offer the advantages of precise temporal and spatial selectivity in cancer treatment, minimizing damage to normal cells while effectively eliminating tumor cells. However, the therapeutic efficacy of phototherapy is always hindered by challenges such as hypoxia and tumor heat resistance. Herein, a pH‐responsive metal‐drug nanocomplex (denoted as PAFH) comprising hypericin (HYP), apigenin (APG), polyvinylpyrrolidone (PVP), and Fe^3+^ is developed to enhance the therapeutic efficacy of PDT and PTT. The PAFH nanocomplex exhibits photothermal properties under 808 nm laser irradiation, which can disassociate in response to the acidic tumor microenvironment and the temperature increase induced by PTT, thereby eventually triggering the on‐site release of APG and HYP. The released APG inhibits the synthesis of heat shock protein HSP‐90, facilitating the PAFH‐mediated PTT to kill tumor cells at mild temperature. Additionally, APG alleviates hypoxia and then regulates the expression of hypoxia‐inducible factor HIF‐1𝛼, increasing cellular oxygen levels to produce singlet oxygen for enhanced HYP‐mediated PDT and inhibiting tumor metastasis. Ultimately, this sophisticated nanosystem represents an advanced strategy to promote PDT and PTT by mitigating tumor hypoxia and counteracting tumor heat resistance, significantly improving therapeutic efficacy for precise cancer therapy.

## Graphical abstract

A pH‐responsive nanocomplex (PAFH) enhances photodynamic (PDT) and photothermal therapy (PTT) by addressing tumor hypoxia and heat resistance. Under laser irradiation, PAFH releases hypericin for singlet oxygen generation and apigenin to inhibit HSP‐90 and alleviate hypoxia. This synergistic approach improves PTT efficiency at mild temperatures and enhances PDT efficacy for precise cancer therapy.

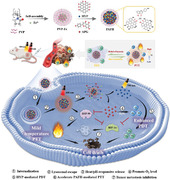

## 1 Introduction

Phototherapy for cancer treatment including photodynamic therapy (PDT) and photothermal therapy (PTT), is recognized for its benefits of precise temporal and spatial selectivity, minimizing damage to normal cells while effectively eliminating tumor cells.^[^ ^1^ ^]^ However, PDT and PTT often fail to suppress distant and metastatic tumor sites due to the tumor hypoxia properties, the development of thermal resistance, and the limited penetration depth of light sources.^[^ ^2^ ^]^ Specifically, PDT is hindered by hypoxia, a prominent feature of the tumor microenvironment due to poor vascular oxygen supply that is not enough to support rapid tumor growth. In order to adapt such conditions, tumors must evolve to survive under hypoxic environments.^[^ ^3^ ^]^ Hypoxia‐inducible factors (HIFs) is a transcription factor of the tumor response to hypoxia, which is highly associated with the decreased Oxygen (O_2_) availability in various solid tumors including breast, colon, renal, etc.^[^ ^4^ ^]^ Additionally, O_2_ is exhausted during PDT, further deteriorating tumor hypoxia in a negative feedback loop. In the case of PTT, thermoresistance may develop due to the activation of cytoprotective pathway by stimulating the expression of heat shock proteins (HSPs). HSP‐90 is a stress‐sensitive protective substance secreted by tumor cells in response to any cell damage, particularly fatal damage.^[^ ^5^ ^]^ HSP‐90 is upregulated when temperature is higher than 38.5 °C to protect the cancer cell from stress‐induced damage, thus weakening the PTT efficacy.^[^ ^6^ ^]^ Combining PDT and PTT offers a promising strategy to address the limitations of monotherapy. Recent studies have demonstrated successful outcomes through the development of various nanomaterials to integrate these therapeutic modalities.^[^ ^7^ ^]^ Specifically, PTT can promote blood circulation, increasing O_2_ levels in tumors and thereby boosting the production of singlet oxygen by PDT. Meanwhile, the reactive oxygen species (ROS) generated during PDT can attack the HSPs, preventing the development of thermoresistance. Also, ROS increases the permeability of tumor cell membrane, enhancing drug delivery and making cells more sensitive to PTT. By incorporating molecules that alleviate hypoxia and inhibit the expression of HSPs, the therapeutic efficacy of combined PDT and PTT can be significantly improved.^[^ ^8^ ^]^

Natural compounds, owing to their inherent origin, represent a major source for drug discovery. Apigenin (APG), a natural plant flavone found in various plants, fruits, and vegetables, has demonstrated anti‐cancer effects across a wide range of tumors by inducing cell apoptosis, triggering autophagy, modulating the cell cycle, and inhibiting cancer cell migration and invasion. Additionally, APG has been found to inhibit the expression of HIF‐1α and HSP‐90, showing the potential to alleviate tumor hypoxia, mitigate oxygen deficiency, and facilitate mild hyperthermia.^[^ ^9^ ^]^ These properties make APG a potential active pharmaceutical ingredient to simultaneously enhance the efficacy of photosensitizers in PDT and photothermal agents in PTT, thus addressing the current challenges of phototherapy. On the other hand, hypericin (HYP) has proven to be an effective natural photosensitizer for treating various cancers.^[^ ^10^ ^]^ Despite the clinical potential of APG and HYP, the effective applications of both compounds are hindered by their poor water solubility and low intestinal absorption, which restrict their efficacy against tumors.^[^ ^11^ ^]^ Encapsulating both drugs into various drug delivery systems such as micelles may help to overcome these limitations, broadening their applications and maximizing their therapeutic efficacies.^[^ ^12^ ^]^ Metal‐drug complexes such as metal‐polyphenols are formed through the coordination interactions between the carbonyl and hydroxyl groups of drug molecules and metal ions like iron, which have been extensively studied for various biomedical applications, including anticancer and anti‐inflammation therapies. Moreover, such nanocomplexes also enable intracellular drug delivery, potentially solving the drawbacks of HYP and APG. In addition, these complexes always have strong absorbability in the visible to near infrared (NIR) region and typically exhibit photothermal properties by converting the NIR light into heat, making it suitable for PTT.^[^ ^13^ ^]^ By integrating a photosensitizer into these complex systems, combined PDT and PTT can be achieved, thus enhancing the therapeutic efficacy of cancer treatment.

Herein, a pH‐responsive metal‐drug nanocomplex comprising HYP, APG, polyvinylpyrrolidone (PVP), and Fe^3+^ was developed by using microfluidic device to achieve combined PDT and PTT (denoted as PAFH). In this system, metal‐drug nanocomplex PAFH dissociates under tumor acidic microenvironment and localized temperature increases, releasing metal ions, APG and HYP in situ, thereby exhibiting a precise controlled‐release property. Then, the released APG suppresses the expression of HSP‐90, counteracting tumor heat resistance for low‐temperature PTT induced by PAFH. Moreover, APG alleviates hypoxia by increasing cellular oxygen content and lowers the expression of HIF‐1α, thus promoting singlet oxygen generation for enhanced HYP‐mediated PDT and inhibiting tumor metastasis and invasion. Furthermore, the localized heat induced by PTT improves the O_2_ supply by increasing blood flow, thus boosting the therapeutic efficacy of PDT.^[^ ^14^ ^]^ On the other hand, ROS produced during PDT can attack the HSP‐90, thereby facilitating mild PTT.^[^ ^15^ ^]^ Overall, this system combines PDT and PTT to collectively inhibit tumor growth, aiming to mitigate tumor hypoxia and counteract tumor heat resistance, offering an advanced strategy for precise cancer therapy.

## 2 Results and Discussion

### 2.1 Preparation and Characterization of PAFH Nanocomplex

**Figure 1:**
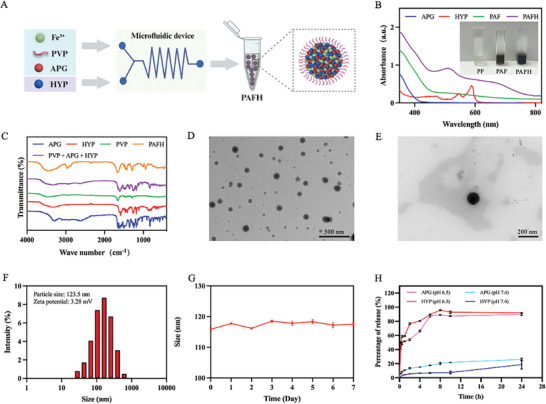
Characterization of PAFH formulations. A) Scheme for PAFH nanocomplex preparation. B) UV–vis absorbance spectra of APG, HYP, PAF, and PAFH nanocomplex. Inset: appearance of PF, PAF, and PAFH. C) FT‐IR spectra of APG, HYP, PVP, physical mixture and PAFH nanocomplex. TEM image of D) PAFH (Scale bar: 500 nm) and E) induvial PAFH (Scale bar: 200 nm). F) Size distribution and zeta potential of PAFH. (G) Stability of PAFH during storage at 4 °C (mean ± SD, *n* = 3). H) In vitro APG and HYP release profile from PAFH under different conditions (mean ± SD, *n* = 3).

PAFH was prepared using a microfluidic system, as illustrated in **Figure** **1**A. First, PVP@Fe^3+^ (denoted as PF) was formed by mixing PVP and Fe^3+^ ions with gentle stirring, which was a colorless aqueous solution. After the subsequent addition of APG, the color of the solution turned to brown, suggesting the complexation between APG and Fe^3+^ (denoted as PAF). The obtained PAF metal‐phenolic nanocomplex was then coordinated with HYP to form the final black‐colored PAFH nanocomplex (Figure 1B). UV spectroscopy was performed to confirm the coordination between Fe^3+^, APG, and HYP. The broad absorption band observed at wavelengths of 500–800 nm in PAFH indicated the complexation between the phenolic drugs and Fe^3+^, so that some of the characteristic peaks of HYP disappeared. In addition, the broad and red‐shifted bands indicated the photothermal properties of the nanocomplex (Figure 1B). The formation of PAFH was further verified by IR analysis (Figure 1C), the characteristic peaks of physical mixture of HYP, APG and PVP showed differences to those of the PAFH nanocomplex, indicating the coordination of HYP and APG with metal ions to form the nanosystem. According to the transmission electron microscopy (TEM) images, PAFH complexes were spherical in shape and distributed evenly in the aqueous media (Figure 1D,E). The ratio of PVP, HYP, APG, and Fe^3+^ was optimized by evaluating the prepared particle size and polydispersity index (PDI) (Figure S1 and Table S1, Supporting Information). Dynamic light scattering (DLS) analysis indicated that the particle size of the obtained PAFH was ≈123.5 ± 6.8 nm, with PDI ≈0.236 ± 0.040 and zeta potential of PAFH was determined to be positive with +3.28 mV (Figure 1F). Furthermore, the particle size remained stable during storage at 4 °C for at least 7 days, suggesting good stability of nanocomplex (Figure 1G). The drug loading efficiencies of APG and HYP in PAFH were determined to be 3.71% and 3.62% using HPLC, respectively. The in vitro release behavior of HYP and APG was also investigated, as shown in Figure 1H. In PBS solution at pH 7.4, the nanocomplex remained stable, releasing only a small percentage of HYP and APG. However, in PBS with pH 6.5, the drugs released rapidly from the nanocomplex, with approximate 85 to 90% cumulative release rate, indicating the pH‐triggered release of the PAFH nanocomplex.

### 2.2 Photothermal Properties of PAFH

**Figure 2:**
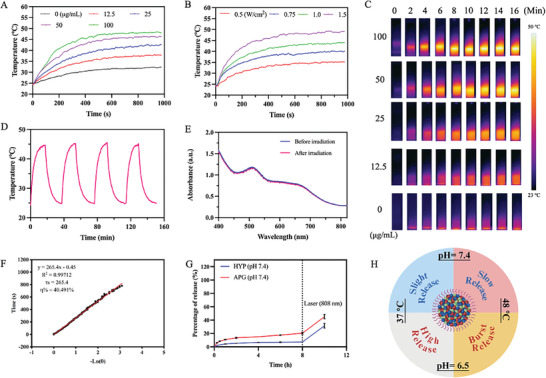
Photothermal performance of PAFH. A) Temperature rising curves of PAFH at different concentrations. B) The relative temperature curves of 100 µg mL⁻^1^ of PAFH solution under 808 nm laser irradiation with different power densities. C) Representative thermal images of PAFH upon 808 nm laser irradiation (1.5 W cm⁻^2^). D) Four cycles of temperature variation in 100 µg mL⁻^1^ of PAFH upon repeated 808 nm laser on/off (1.5 W cm⁻^2^). E) UV spectrum of PAFH solution before and after 10 min of light irradiation (1.5 W cm⁻^2^). F) Curve of heat conversion efficiency. G) In vitro drug release profile of APG and HYP from PAFH before and after 30 min of 808 nm laser irradiation (1.5 W cm⁻^2^, mean ± SD, *n* = 3). H) Summary of release conditions.

As shown in Figure 1B, PAFH exhibited broad absorptions in the region of 500–810 nm due to the coordination between APG and HYP with Fe^3+^ in PAFH. According to previous findings,^[^ ^16^ ^]^ PAFH is likely to exhibit photothermal properties under light irradiation at 808 nm. As illustrated in **Figure** **2**A–C, the temperature of PAFH solution upon laser treatment showed a time‐, drug concentration‐ and laser power density‐dependent. Specifically, the temperature of a 100 µg mL⁻^1^ PAFH solution reached a maximum temperature over 45 °C after 10 min of laser irradiation at a power density of 1.5 W cm⁻^2^, which is a temperature suitable for mild‐temperature PTT. In addition, the photothermal performance of PAFH formulations with different iron content was evaluated. Results showed that increasing iron content (APG: Fe^3+^: HYP ratio of 1:10:1) did not significantly enhance photothermal properties (Figure S2, Supporting Information). The photothermal stability of PAFH was also evaluated, as shown in Figure 2D. The heat generating ability did not show significant changes after four cycles of light irradiation. Additionally, the UV spectrum of PAFH did not alter before and after irradiation (Figure 2E). The photothermal conversion efficiency (η) of PAFH was calculated following the methodology outlined in a previously published study.^[^ ^17^ ^]^ The heat conversion efficiency of PAFH was determined to be 40.491% (Figure 2F). In addition, Figure 2G revealed that the release rates of HYP and APG accelerated upon heating, proving the heat‐triggered release manner of PAFH and the drug release profiles of PAFH under different conditions are illustrated in Figure 2H. Collectively, these optimistic results indicated that PAFH held promise as a material with controlled‐release and photothermal properties.

### 2.3 Anti‐Cancer Effect of PAFH Formulations

**Figure 3:**
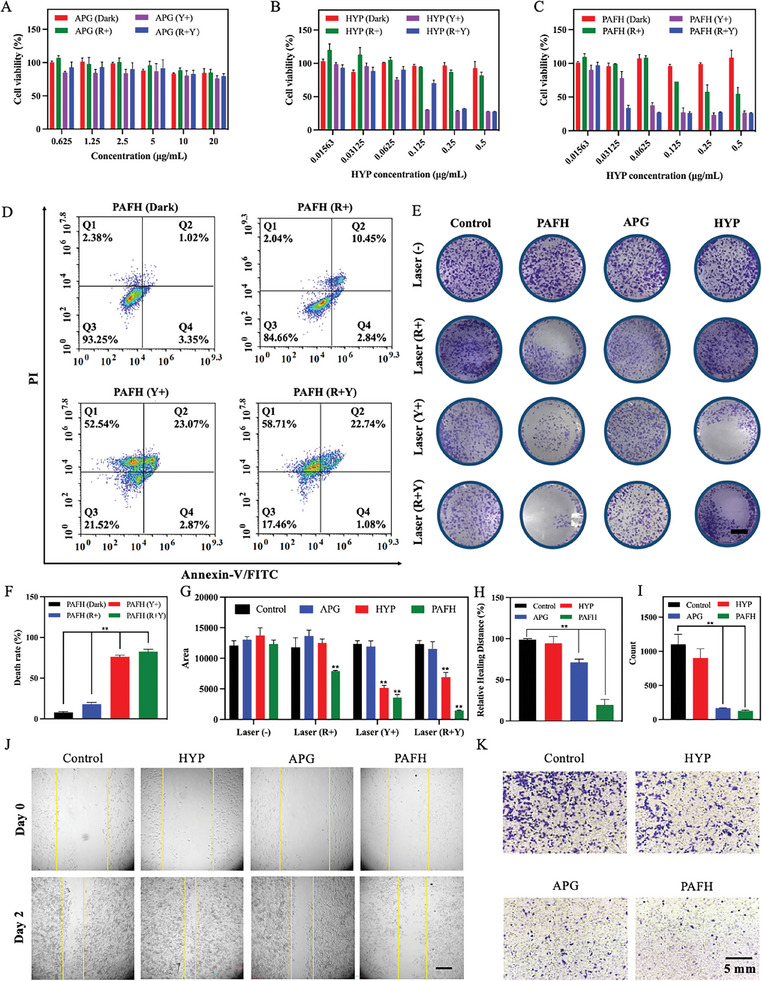
In vitro anti‐cancer effect of APG and HYP formulations on 4T1 cells. MTT assay of dark toxicity and phototoxicity of A) APG, B) HYP, and C) PAFH. D,E) Cells apoptosis analysis by flow cytometry (mean ± SD, *n* = 3). F,G) The colony formation of 4T1 cells treated with PAFH, APG, and HYP with or without laser irradiation (mean ± SD, *n* = 3, scale bar: 5 mm). H) Quantitative analysis and I) representative images of wound healing assay after treatment with PAFH, APG, and HYP, scale bar: 200 µm. The yellow lines define the metastasis distances (mean ± SD, *n* = 3). J) Quantitative analysis and K) representative images of Transwell cell migration after treatment with PAFH, APG, and HYP, scale bar: 5 mm, (mean ± SD, *n* = 3). *p < 0.05, **p < 0.01.

The 4T1 cancer cell line was chosen to evaluate the PDT effect of PAFH due to its superficial lesions. MTT assays were conducted to assess the phototoxicity and dark toxicity of the various components within the iron‐phenolic nanocomplex. From **Figure** **3**A–C, it can be observed that free APG, HYP, and PAFH did not exhibit significant cytotoxicity within the tested concentration range in the absence of light treatment. Upon yellow light irradiation (590 nm, Y+), APG did not show any phototoxicity across the tested concentrations, while HYP and the fabricated PAFH revealed PDT effects with a concentration‐dependent manner, confirming the ability of HYP acting as a photosensitizer to induce PDT effects under light‐excited conditions with specific wavelength (Figure 3B,C). In addition, PAFH also exhibited toxicity upon exposure to an 808 nm light source (R+) while HYP did not, confirming the photothermal properties of PAFH (Figure 3B,C). Notably, PAFH showed the strongest cell inhibition effect upon 808 nm light irradiation, followed by 590 nm laser irradiation (R+Y). The IC50 of PAFH was determined to be 0.023 µg mL⁻^1^, which was much lower than that of free HYP (≈0.18 µg mL⁻^1^). Theoretically, Fe^3+^ within PAFH can contribute to the chemodynamic therapy by catalyzing the Fenton reaction to produce ROS under acidic conditions, enhancing the anti‐tumor effect.^[^ ^18^ ^]^ However, due to the low concentration of PAFH required for PDT, the concentration of Fe^3+^ was insufficient to induce ROS via Fenton reaction (Figure S3, Supporting Information). Nevertheless, Fe^3+^ can form coordination complex with APG and HYP, functioning as a linker to enhance the water solubility of APG and HYP, playing an important role in nanocomplex formation. Additionally, the cell death induction by PAFH was also determined. As shown in Figure 3D,E, PAFH treated with 808 nm and 590 nm dual‐laser irradiation had the highest cell death rate and the lowest cell viability (17.46%), which was consistent to the MTT results. These findings indicated that the PAFH combined PTT and PDT significantly induced 4T1 tumor cell death. Colony formation experiments effectively reflected the reproductive capacity of tumor cells after various treatments, as shown in Figure 3E,G. Under dark conditions, the colony formation ability of 4T1 cells treated with various formulations was not affected, which was also similar to the MTT results. After light irradiation, compared to the control group and free drug treatment groups, PAFH showed the least colony‐forming ability and performed the best combined PDT and PTT efficacy, further validating the MTT results. From the experimental results of MTT assay, colony formation study and cell death analysis, PAFH exhibited the therapeutic effects at such low concentrations, which may be due to the enhancement of solubility and modulation of tumor microenvironment, such as reliving hypoxia and inhibition of HSP‐90 synthesis in the presence of APG. Furthermore, the abilities of suppressing cancer cell migration and invasion were also evaluated. In the wound healing assay (Figure 3H,J), APG significantly inhibited the migration and invasion rate of cancer cells, potentially due to the regulation of genes or proteins involved in these processes, such as HIF‐1𝛼, PI3K, GSK‐3β, and MMP2.^[^ ^19^ ^]^ Similar results were observed in the Transwell experiment, as seen in Figure 3I,K. Free APG had the ability to slow down the migration of 4T1 cells, while free HYP did not possess this ability. Interestingly, forming nanocomplex further enhanced the ability of APG to inhibit cell migration. Furthermore, the impact of various formulations on cell migration was compared to the conditions with laser exposure. A much lower concentration of HYP‐containing formulations (0.0625 µg mL⁻^1^) was selected for experiment upon light irradiation due to the PDT and PTT effects induced by HYP and PAFH, respectively. As shown in Figures S4–S7 (Supporting Information), APG maintained its ability to inhibit the cancer cell migration even at relatively low concentrations. Meanwhile, free HYP significantly inhibited cell migration by inducing cancer cell death through PDT pathway, while PAFH further promoted the inhibition effects mediated by both APG and HYP.

### 2.4 Relief of Hypoxia by PAFH and Inhibit HSP‐90 Protein

**Figure 4:**
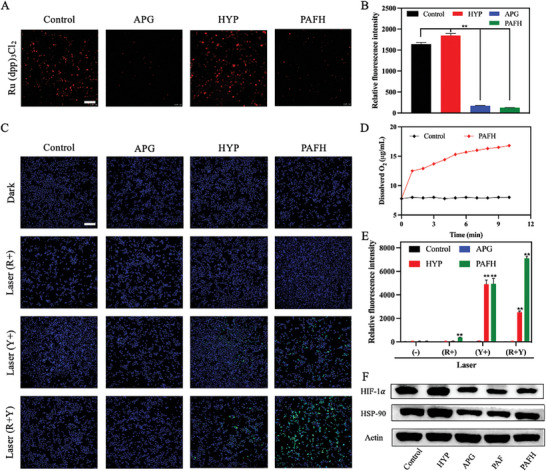
Relief of hypoxia. A) Fluorescence images and B) quantitative analysis of oxygen content in 4T1 cells after various treatments by using Ru(dpp)_3_Cl_2_ probe, scale bar: 200 µm (mean ± SD, *n* = 3). C) Fluorescence images and D) quantitative analysis of intracellular ROS induced by APG and HYP containing formulations using DCFH‐DA probe, scale bar: 200 µm (mean ± SD, *n* = 3). E) O_2_ production by PAFH in the presence of H_2_O_2_. F) Western blot analysis to evaluate the HIF‐1α and HSP‐90 expressions in 4T1 cells after treatment, *p < 0.05, **p < 0.01.

As mentioned above, the presence of APG in PAFH may alleviate the hypoxia and inhibit HSP‐90 protein synthesis in the tumor microenvironment. Therefore, the hypoxia within 4T1 cells was assessed using the [Ru(dpp)_3_]Cl_2_ fluorescence O_2_ probe.^[^ ^20^ ^]^ As shown in **Figure** **4**A, the control group exhibited significant red fluorescence, indicating a relatively hypoxic tumor microenvironment that could hinder the effectiveness of PDT efficacy. In contrast, treatment with free APG resulted in quenching of red fluorescence, indicating an increase in O_2_ content within the tumor cells. Notably, even after constructing the PAFH nanocomplex, the presence of APG continued to effectively improve the hypoxic conditions in tumor cells, demonstrating that the fabricated drug delivery system can effectively improve the hypoxic environment and further promote HYP‐mediated PDT effects. Quantitative analysis of O_2_ content corroborated the trends observed in the fluorescence images taken by the fluorescence microscope (Figure 4B). Furthermore, the ability of PAFH to produce O_2_ in the presence of H_2_O_2_ was proven as presented in Figure 4D. The DCFH‐DA detection probe was employed to visualize ROS generation in 4T1 cells by HYP‐containing formulations upon light irradiation, with quantitative analysis conducted via flow cytometry. As shown in Figure 4C,E, PAFH exhibited stronger green fluorescence compared to the single HYP treatment group, implying higher ROS levels in 4T1 cells owing to the improvement of tumor hypoxia. Additionally, the effect of lighting sequence on the ROS production was investigated. Results demonstrated that irradiation with red light (808 nm) first produced a larger amount of ROS compared to irradiation with yellow light (590 nm) first (Figure S8, Supporting Information). The expression of HIF‐1α and HSP‐90 proteins was further evaluated by western blot study (Figure 4F). Results showed that under dark conditions, both the APG and the PAFH nanocomplex effectively reduced HIF‐1α protein expression, indicating that both formulations effectively alleviated the hypoxia in the tumor microenvironment. Although PDT always exacerbates the tumor hypoxic environment, APG and PAFH were still able to alleviate this condition following light exposure, demonstrating the potential of APG in enhancing PDT by relieving tumor hypoxia.

### 2.5 Cellular Uptake, Spheroid Penetration and Biodistribution of PAFH

**Figure 5:**
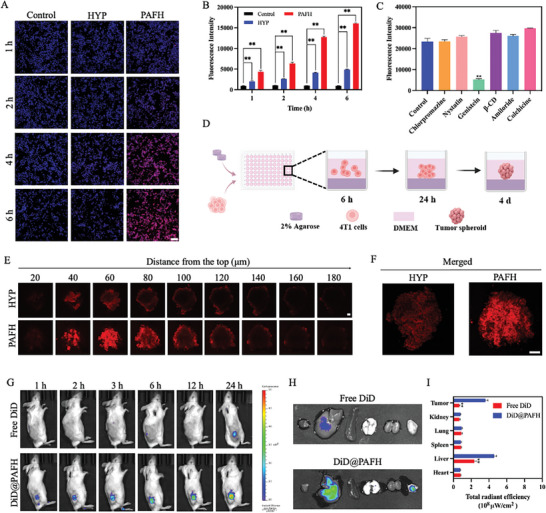
Cellular uptake and tumor penetration. A) Fluorescence images and B) quantitative analysis of 4T1 cells fluorescence intensity after treatment with HYP and PAFH (concentration of HYP: 0.5 µg mL⁻^1^, scale bar: 100 µm, mean ± SD, *n* = 3). C) Mechanism study of cellular uptake (mean ± SD, *n* = 3). D) Scheme of 3D tumor penetration study. E) Representative tumor spheroid images after treating with HYP and PAFH (concentration of HYP: 0.5 µg mL⁻^1^, scale bar: 20 µm). F) Merged images of tumor spheroid (scale bar: 20 µm). G) Biodistribution of free DiD and DiD@PAFH at different time points. H) Ex vivo images of major organs and tumor after 24 h of administration. I) Fluorescence intensity of free DiD and DiD@PAFH in major organs and tumor (mean ± SD, *n* = 3). *p < 0.05, **p < 0.01.

Due to the intrinsic fluorescence property of HYP, no additional dye was required for cellular uptake study. As shown in **Figure** **5**A, the cellular uptake of PAFH increased with the rising concentration of HYP. PAFH displayed stronger red fluorescence compared to the free HYP, indicating that the nano formulation promoted the cellular uptake of payload. In addition, quantitative analysis showed a consistent trend with the observations under the fluorescence microscope, confirming the delivery ability of the iron‐phenolic nanocomplex (Figure 5B). Subsequently, different uptake inhibitors were employed to investigate the uptake mechanism of PAFH. The low cytotoxicity of these inhibitors at their working concentrations was confirmed (Figure S9, Supporting Information), and the results showed that the uptake of PAFH may occur through caveolae‐mediated endocytosis (Figure 5C).^[^ ^21^ ^]^ On the other hand, tumor spheroid of 4T1 cells was incubated for penetration study. As shown in Figure 5E,F, PAFH displayed a stronger red fluorescence intensity in the spheroid core compared to the free HYP, demonstrating the superior tumor penetration of PAFH. These findings clearly confirmed that the fabricated drug delivery system significantly improved the cellular uptake and spheroid penetration of HYP and APG, which can be attributed to the enhanced permeability and retention (EPR) effect. To further investigate the biodistribution profile of PAFH, a 4T1 mouse xenograft model was established. Following intravenous injection of free DiD and DiD‐labelled PAFH, fluorescence signals were monitored using IVIS at different time points. As illustrated in Figure 5G, fluorescence of DiD‐labelled PAFH was detected at the tumor site of mice within 1 h post‐injection, and the intensity increased over time, indicating rapid and sustained accumulation of PAFH at the tumor site while minimal fluorescence signal was observed in free DiD group after administration. In addition, ex vivo fluorescence imaging of excised tumors further supported the in vivo biodistribution results (Figure 5H,I). The maximum fluorescence signal intensity was observed in the tumor, surpassing that of major organs, demonstrating that DiD‐labelled PAFH exhibited tumor targeting capabilities.

### 2.6 In Vivo Antitumor Effect and Biosafety of PAFH

**Figure 6:**
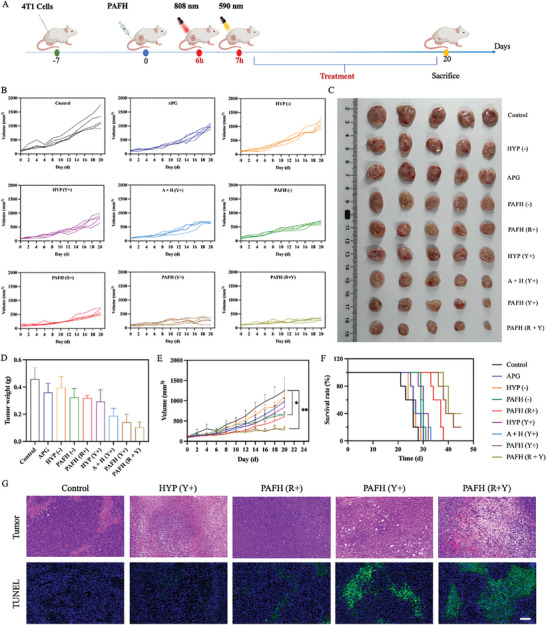
Antitumor efficacy of PAFH in 4T1 tumor‐bearing mice. A) Scheme of in vivo therapy. B) Individual tumor growth curves of the groups of Control, AGP, HYP (−), HYP (Y+), A + H (Y+), PAFH (−), PAFH (Y+), PAFH (R+) and PAFH (R + Y) (*n* = 5). C) The image of excised tumors on day 21 after inoculation. D) The weights of tumors after treatments (mean ± SD, *n* = 5). E) Average tumor growth curves with different treatments (mean ± SD, *n* = 5). F) Survival curves after different treatments (*n* = 5). G) H&E and TUNEL images of tumors of selected treatment groups. Scale bar: 100 µm. *p < 0.05, **p < 0.01.

The therapeutic effect of PAFH in inhibiting tumor growth was evaluated (**Figure** **6**A). After 21 days of treatment, PAFH with dual‐light irradiation showed the most significant antitumor effects (Figure 6B–E). The single use of APG or HYP without light irradiation did not significantly inhibit the tumor growth compared to the control group. Meanwhile, the single use of HYP with 590 nm light irradiation only slightly inhibited tumor growth, which is likely due to the poor solubility and hypoxic tumor microenvironment. Remarkably, the combined use of APG and HYP with 590 nm laser irradiation promoted the antitumor effects compared to either single drug. Importantly, sequential irradiation with red light (808 nm) then followed by yellow light (590 nm) further promoted the antitumor efficacy of PAFH irradiated with yellow light (590 nm), providing the therapeutic efficacy of combined PDT and PTT. In addition to its PTT effects, the risen local temperature due to the mild heating may enhance tumor blood flow and elevate O_2_ levels within the tumor microenvironment, thus amplifying the efficacy of PDT. As shown in Figure 6G, H&E and TUNEL staining revealed that PAFH with dual‐light irradiation treatment led to more extensive tumor cell death compared to other treatment groups. Furthermore, the survival period of mice receiving the combined PDT and PTT treatment was significantly prolonged (Figure 6F).

**Figure 7:**
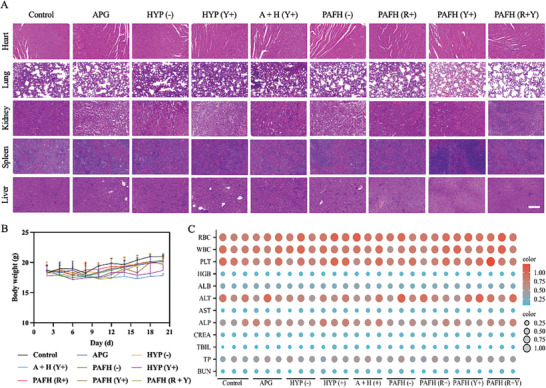
Biosafety evaluation. A) Representative H&E images of heart, lung, kidneys, spleen, and liver after treatments. Scale bar: 100 µm. B) The body weights of mice during treatments (mean ± SD, *n* = 5). C) Complete blood count and serum biochemistry profile for biosafety evaluation.

We also evaluated the biosafety of various APG and/or HYP formulations after treatment. H&E images of major organs indicated treatment with PAFH did not cause tissue damage in the heart, liver, spleen, lungs, or kidney (**Figure** **7**A). In addition, no significant changes in the body weight of mice were observed receiving different treatments (Figure 7B). Blood samples were also collected from mice for routine blood tests and biochemical analyses, which revealed no deviations from the reference standards. Additionally, the serum biochemistry profile confirmed normal liver and kidney functions (Figure 7C). These findings collectively demonstrate the overall safety of PAFH nanocomplex.

## 3 Conclusion

In summary, this study demonstrates the potential of the pH‐responsive iron‐phenolic nanocomplex (PAFH) as an integrated system for realizing combined PDT and PTT. Our findings highlight the ability of APG to alleviate hypoxia and mitigate thermoresistance by downregulating HSP‐90 expression, thereby enhancing the efficacy of HYP‐mediated PDT and PAFH‐mediated PTT. In vitro and in vivo experiments have demonstrated the therapeutic efficacy and biosafety of PAFH for breast cancer treatment. This innovative strategy holds great promise for advancing cancer therapy, offering a targeted and effective treatment option with the potential for clinical translation and significant benefits for patients in the future.

## 4 Experimental Section

### Materials

Ferric chloride (FeCl_3_) was purchased from Damao Chemical Reagent Factory (Tianjin, China). Apigenin (APG) was bought from Innochem Science & Technology Co., Ltd (Beijing, China). Hypericin (HYP) was provided by Yirui Biotechnology Co. Ltd (Chengdu, China). Glutathione (GSH), methanol, and dimethyl sulfoxide (DMSO) were purchased from Aladdin Bio‐Chem Technology Co., Ltd (Shanghai, China). 3‐[4,5‐dimethylthiazol‐2‐yl]‐2,5 diphenyl tetrazolium bromide (MTT) was obtained from Sigma‐Aldrich (Saint Louis, United States). 6.5 mm Transwell with 8.0 µm Pore Polycarbonate Membrane Inserts was obtained from Corning (New York, United States). Luminescent oxygen sensor [Ru(dpp)_3_]Cl_2_ was purchased from MaokangBio (Shanghai, China). DCFH‐DA probe and Hoechst 33342 were obtained from Beyotime Biotechnology (Shanghai, China).

### Cell Line

Murine mammary carcinoma 4T1 cells were obtained from American Type Culture Collection (Rockville, United States) and cultured in Dulbecco's Modified Eagle Medium supplemented with 10% fetal bovine serum and 1% penicillin‐streptomycin (Gibco, Massachusetts, United States) in an incubator at 37 °C maintaining 5% CO_2_.

### Preparation and Characterization of PAFH

First, 80 mg of PVP was dissolved in 4 mL of water. Then 500 µL of FeCl_3_ (10 mg mL⁻^1^) was dropwise added into PVP solution under vigorous stirring. After 30 min of stirring, 1 mL of APG (1 mg mL⁻^1^) DMSO solution was dropwise added into the PF solution under vigorous stirring and the solution was incubated in dark environment for 30 min to yield the PAF. PAFH nanoparticles were prepared using a two‐channel microfluidic system. PAF NPs solution and HYP solution were injected into the microfluidic system at a flow ratio of 5:1 via polypropylene syringes with a size of 10 and 1 mL respectively. After injection, the obtained nanoparticles were collected, and the organic solvent was removed by ultrafiltration. The particle size and zeta potential of PAFH were determined by a Zetasizer (Malvern, UK). The morphology of PAFH was observed by TEM.

### The Photothermal Properties of Nanoparticles

The in vitro photothermal properties of PAFH were evaluated with Fortic 225 IR thermal camera (Fotric, China) and AnalyzIR software by measuring the heating curves of distilled water and PAFH under 808 nm laser irradiation (1.5 W cm⁻^2^) at different concentrations (0, 12.5, 25, 50, 100 µg mL⁻^1^).

### In Vitro Drug Release of APG and HYP

In vitro release rates of APG and HYP from PAFH were examined by dialysis. First, 1 of mL PAFH samples were placed in dialysis bags (MWCO 3500 Da) and immersed into release media (30% methanol, 1% SDS, and 69% PBS) with pH 7.4 or pH 6.5. Each group of release media was then incubated in a shaker at 37 °C with shaking for 24 h. 1 mL of release media was withdrawn at specific time points (0 min, 15 min, 30 min, 1 h, 2 h, 4 h, 6 h, 8 h, 10 h, and 24 h) to determine the amount of drug released, and the same volume of the corresponding fresh release media was immediately replenished thereafter. The percentage of drug release formula is listed below: Percentage of drug release = amount of drug release/total amount of drug × 100%

### Phototoxicity and Cytotoxicity Studies of HYP, APG, and PAFH Formulations

MTT assay was performed to assess the in vitro cytotoxicity and phototoxicity of PAFH formulations against 4T1 cells. 5 × 10^3^ 4T1 cells were cultured onto 96‐well plate in an incubator for 24 h. Then, the medium was replaced with DMEM containing free HYP, APG, and PAFH. For phototoxicity studies, cells were received light irradiation with 808 nm or/and 590 nm wavelength at an intensity of 1.5 W cm⁻^2^ or/ and 4.72 mW cm⁻^2^, respectively, after 4 h of incubation. For cytotoxicity evaluations, the cell did not receive any laser treatment during this period. After laser exposure, MTT containing DMEM medium was added to replace the drug containing medium for further 4 h of incubation. Then, MTT medium solution was gently removed, followed by adding 100 uL of DMSO. Finally, the absorbance at 490 nm was detected by SpectraMax microplate reader (Molecular Devices, SJ, CA).

### Intracellular ROS Detection

A density of 5 × 10^4^ 4T1 cells were seeded onto each well of a 24‐well plate. After 24 h, cultured medium was substituted by DMEM medium containing free HYP, APG, and PAFH with drug (HYP and/or APG) concentration of 0.0625 µg mL⁻^1^ in irradiation group and 0.5 µg mL⁻^1^ in non‐irradiation group for 4 h incubation. For light irradiation, cells were irradiated by light source with 808 nm or/and 590 nm wavelength at an intensity of 1.5 W cm⁻^2^ or/ and 4.72 mW cm⁻^2^, followed by DCFH‐DA probe staining for 30 min. Then, the cells were washed with PBS three times before capturing the images by using a DMI8 fluorescent microscope (Leica, Wetzlar, Germany).

### Cell Apoptosis Study

5 × 10^5^ 4T1 cells were seeded in 6‐well plates and were treated with PAFH formulations at the equiv. HYP concentration of 0.0625 µg mL⁻^1^, and given or not given light source irradiation with 590 nm and/or 808 nm wavelength for 10 min, respectively (without light treatment, refer to as PAFH (‐)), PAFH (yellow light treatment, refer to as PAFH(Y+)) and PAFH (red and yellow light treatment, refer to as PAFH (R+Y)). After 24 h of incubation, the cells were collected by trypsinization, washed twice with cold PBS. Then, cells were incubated with Annexin V‐FITC and propidium iodide (PI) according to the manufacturer's instructions. After that, the samples were analyzed using a flow cytometer, and the data were processed to quantify the percentage of cells in each quadrant.

### Colony Formation Assay

A density of 500 4T1 cells were seeded onto a 24‐well plate and incubated overnight. Then, cultured medium was replaced by DMEM medium containing free HYP, APG, and PAFH with drug (HYP and/or APG) concentration of 0.0625 µg mL⁻^1^ for 24 h. After that, cells were irradiated by a light source with 808 nm and/or 590 nm wavelength at an intensity of 1.5 W  cm⁻^2^ and/or 4.72 mW cm⁻^2^. Next, drug containing medium was discarded and fresh DMEM was added for 10 days of incubation. Finally, 4% paraformaldehyde was employed to fix the cells, which were then stained by crystal violet.

### Wound Healing Assay

Wound healing assay was performed to assess the migratory ability of 4T1 cells after treated with PAFH. 4T1 cells were seeded onto a 24‐well plate with cell density of 5 × 10^4^ per well in DMEM. A cross was scratched in each well and DMEM containing free HYP, APG, and PAFH with drug (HYP and/or APG) concentration of 0.5 µg mL⁻^1^ was added in each well and further incubated for 48 h. Cell migration images were taken by DMI8 inverted fluorescence microscope.

### Cellular Uptake

4T1 cells were seeded and cultured 24 h at a density of 5 × 10^4^ cells per well in a 24‐well plate. 0.5 µg mL^−1^ of HYP contained formulation was added to the cells for internalization. At various time points after incubation, cells were observed by a DMI8 fluorescent microscope, and the fluorescence intensity of cells was quantified using a flow cytometer.

### Penetration of the 3D Tumor Spheroids

Tumor spheroids were established in advance to assess the penetration ability of different formulations. In brief, 2% (w/v) agarose serum free DMEM medium was used to pre‐coated 96‐well plates (80 uL for each well). After solidification, 4T1 cell was seeded onto each well with intensity of 5 × 10^3^ and further cultured at 37 °C incubator for 4 d to form the tumor spheroids. Uniform and compact spheroids were transferred to confocal dishes and fresh DMEM containing free HYP, APG, and PAFH with drug (HYP and/or APG) concentration of 0.5 µg mL⁻^1^ was added for 24 h incubation. Finally, Z‐stack images of the 3D spheroids were obtained by TCS SP8 MP confocal laser scanning microscope (Leica, Wetzlar, Germany).

### Western Blot

The protein levels in 4T1 cells were assessed through western blot analysis. Briefly, cells were lysed by 150 µL of RIPA (containing PMSF). The total amount of extracted proteins was quantified using the BCA protein assay kit (A53226, Thermo Fisher Scientific) and denatured in 5x loading buffer at 95 °C for 10 min. 20 µg of protein was loaded on to a 10% acrylamide gel and transferred to a PVDF membrane followed by blocking with 5% skim milk in TBST. Then the membranes were incubated with primary antibodies (HIF‐1α, HSP‐90, and actin) overnight at 4 °C, followed by incubation with horseradish peroxidase‐conjugated secondary antibodies. The protein was visualized using an enzymatic chemiluminescence kit (Pierce, Rockford, IL, USA) and imaged with a gel documentation system (ChemiDoc MP, Bio‐Rad, USA).

### Cell Migration Assay

24‐well plate fortified with Transwell membrane filters (8 µm, Corning Inc.) were applied. 5 × 10^4^ 4T1 cells were resuspended in serum‐free medium containing APG and HYP formulations, inoculated into upper chambers for 12 h. The migrated cells were stained with crystal violet dye and observed under a microscope.

### Animals

Female BALB/C mice (5–6 weeks) were purchased from Gunagdong Medical Laboratory Animal Center (Guangzhou, China). Experiment protocols were approved by the Academic Ethics Committee of The Zhuhai Campus of Zunyi Medical University (No. ZHSC‐2‐[2024]14). To inoculate the subcutaneous 4T1 model, 5 × 10^5^ 4T1 cells suspended in 100 µL PBS were injected into the back of each mouse.

### Biodistribution of PAFH In Vivo

When the tumor volume reached ≈250 mm^3^, free DiD and DiD‐labeled PAFH were administered intravenously (10 µg per mice), and the biodistribution was monitored at various time points using an IVIS imaging system. At the end of the study, major organs and tumor of each mouse were collected for fluorescence imaging.

### In Vivo Antitumor Effect of PAFH

When the tumor volume reached ≈100 mm^3^, the mice were randomly divided into nine groups (*n* = 5) with following treatments: control, free APG, free HYP (without light treatment, refer to as HYP (‐)), free HYP (yellow light treatment, refer to as HYP (Y+)), APG + HYP (yellow light treatment, refer to as (A + H (Y+)), PAFH (without light treatment, refer to as PAFH (‐)), PAFH (yellow light treatment, refer to as PAFH(Y+)), PAFH (red light treatment, refer to as PAFH (R+)), and PAFH (red and yellow light treatment, refer to as PAFH (R+Y)). The dosage of HYP of APG was 5 mg per kg of body weight and administered via the tail vein injections every other day for a total of 10 times. Light irradiation (30 W, 15 mins) was applied after 6 h of administration. The body weight and tumor volume of the mice were recorded every other day after treatment. Tumor volume was calculated by following formula: 1/2 × length × width × width, where the length and width were measured by the long and short axis diameter of tumor, respectively. On day 21, the mice were euthanized. Then, tumors, major organs, and peripheral venous blood were collected for analysis.

### Biosafety Evaluation

Major organs were dissected and embedded in paraffin, followed by H&E staining for pathological observation using a microscope. Blood samples from mice were collected for serum biochemistry analysis. Whole blood count and blood biochemical index analysis were performed by collecting blood plasma from each group and centrifuging it at 3000 RPM for 10 min.

### Statistical Analysis

All experiments were repeated at least 3 times, and the results are presented as the mean ± SD without specifying. Student's t test was employed to compare the differences between two independent groups. Statistical analyses were performed using GraphPad Prism 8. Statistical significance is expressed as *p < 0.05, **p < 0.01, and ***p < 0.001.

## Conflict of Interest

The authors declare no conflict of interest.

## Supporting information



Supporting Information

## Data Availability

The data that support the findings of this study are available in the supplementary material of this article.
