# Supplementary material for: Active Iron‐Drug Nanocomplexes Improve Photodynamic and Photothermal Cancer Therapy by Mitigating Tumor Hypoxia and Counteracting Tumor Heat Resistance
Source: Adv Healthc Mater. 2025 Feb 23;14(9):2404485. doi: 10.1002/adhm.202404485 (PMC11973942; doi:10.1002/adhm.202404485)
Supplement: Supplementary file 1 — Supporting Information [file ADHM-14-0-s001.docx]

Active iron-drug nanocomplexes improve photodynamic and photothermal cancer therapy by mitigating tumor hypoxia and counteracting tumor heat resistance

Yuying Yin^1^, Ka Hong Wong^1^, Liewei Wen ^3^, Meiwan Chen^1,2^*

1. State Key Laboratory of Quality Research in Chinese Medicine, Institute of Chinese Medical Sciences, University of Macau, Macau SAR, China.

2. MoE Frontiers Science Center for Precision Oncology, University of Macau, Macau SAR, China

3. Guangdong Provincial Key Laboratory of Tumor Interventional Diagnosis and Treatment, Zhuhai People's Hospital (Zhuhai Clinical Medical College of Jinan University), Jinan University, Zhuhai, Guangdong 519000, China

E-mail: [mwchen@um.edu.mo](mailto:mwchen@um.edu.mo)

**Supporting Figures**


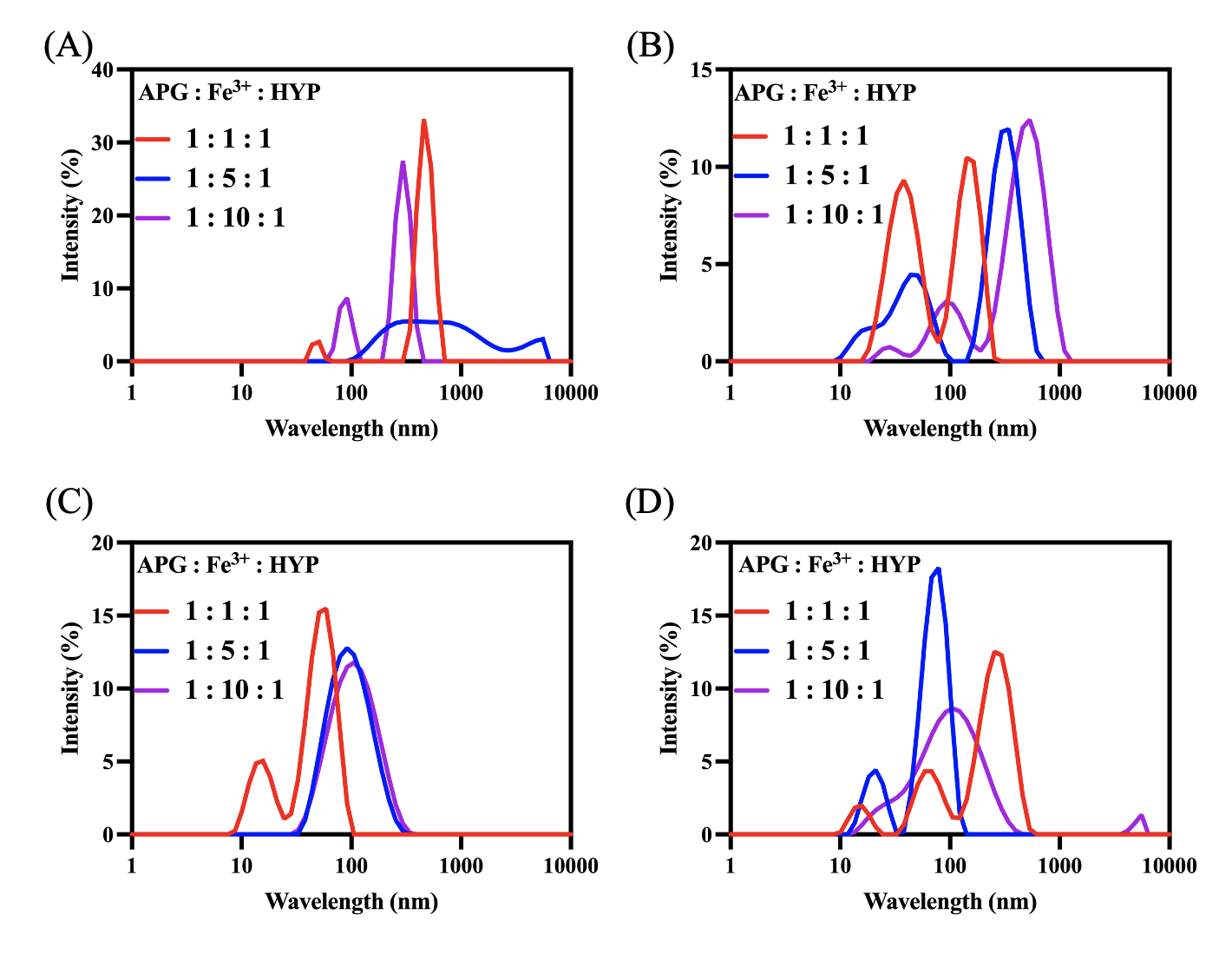


**Figure S1**. Size distribution of PAFH at different composition ratios. A) Size distribution of PAFH containing 20 mg of BSA at different drug/metal ratios. Size distribution of PAFH containing B) 10 mg, C) 20mg, D) 40 mg of PVP at different drug/metal ratios.


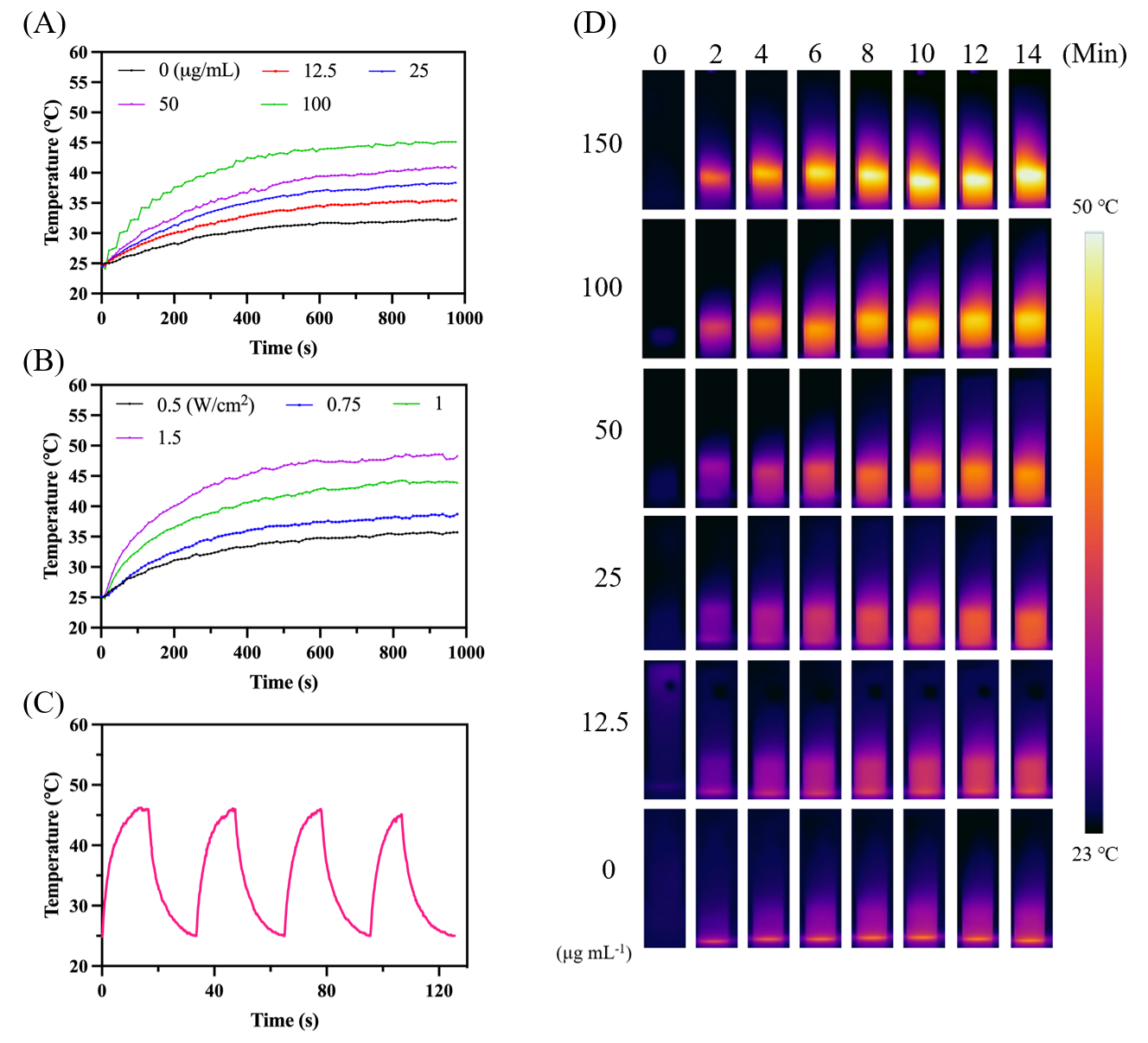


**Figure S2. Photothermal performance of PAFH10.** Temperature curves of PAFH10 at different A) concentrations and B) power densities upon laser irradiation (100 µg/mL). C) Four cycles of temperature variation. D) Representative thermal images of PAFH10 upon laser irradiation.


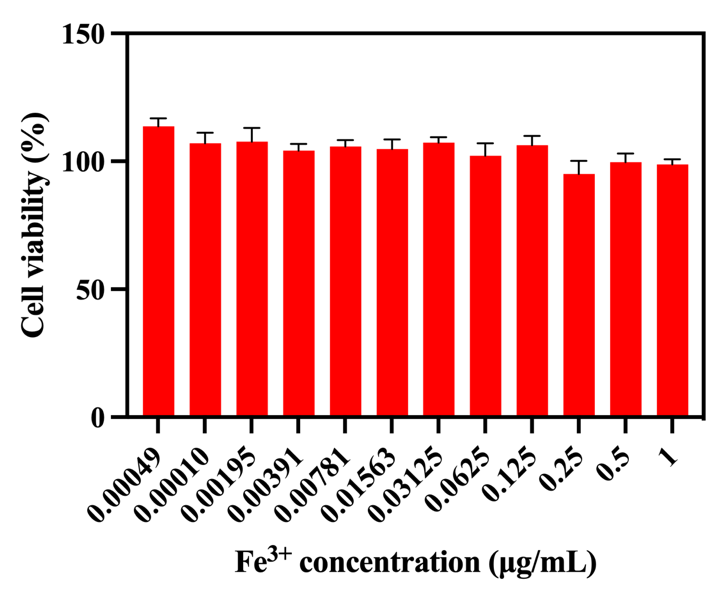


**Figure S3. *In vitro* anti-cancer effect of PAFH on 4T1 cells.** MTT assay of different concentration of Fe^3+^ presented in PAFH (mean ± SD, n = 3).


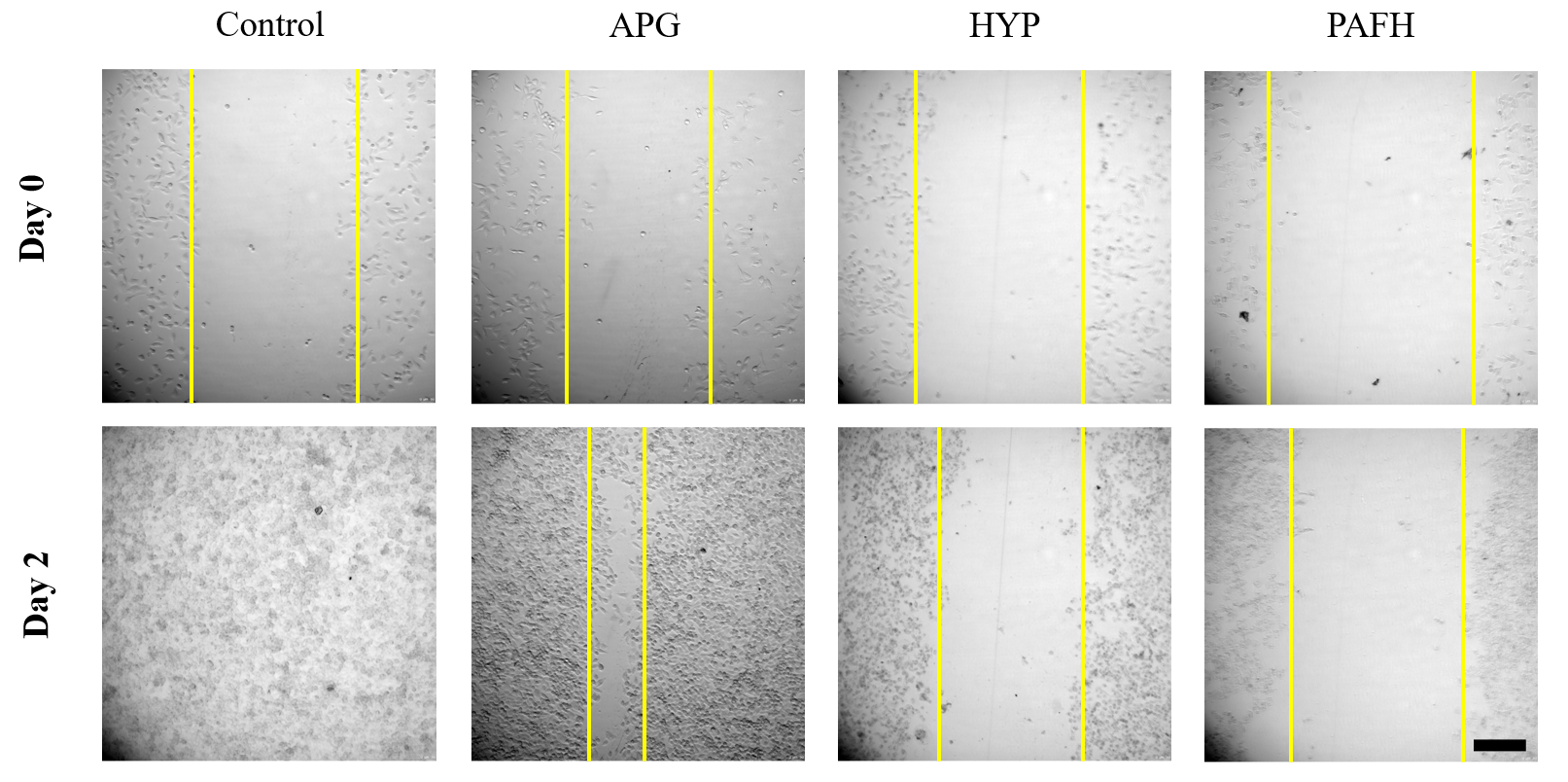


**Figure S4**. Representative images of wound healing assay after treatment with PAFH, APG and HYP with R+Y laser irradiation, scale bar: 200 μm.


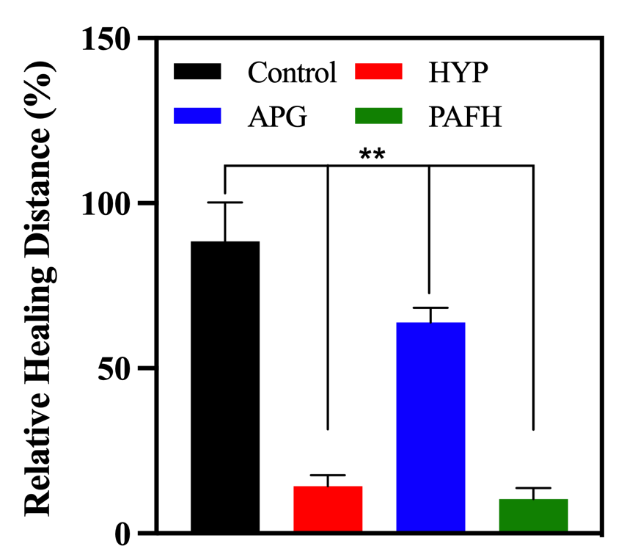


**Figure S5**. Quantitative analysis of wound healing assay after treatment with PAFH, APG and HYP with R+Y laser irradiation (mean ± SD, n = 3).


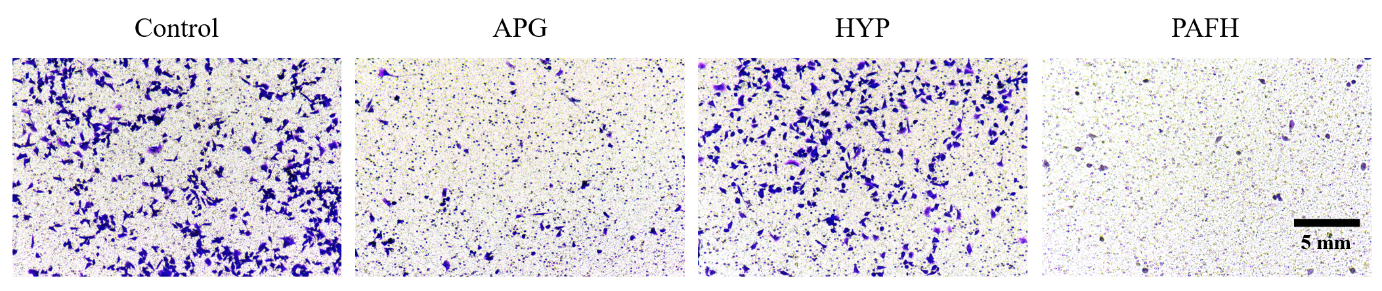


**Figure S6**. Representative images of Transwell cell migration after treatment with PAFH, APG and HYP with R+Y dual laser irradiation, scale bar: 5 mm.


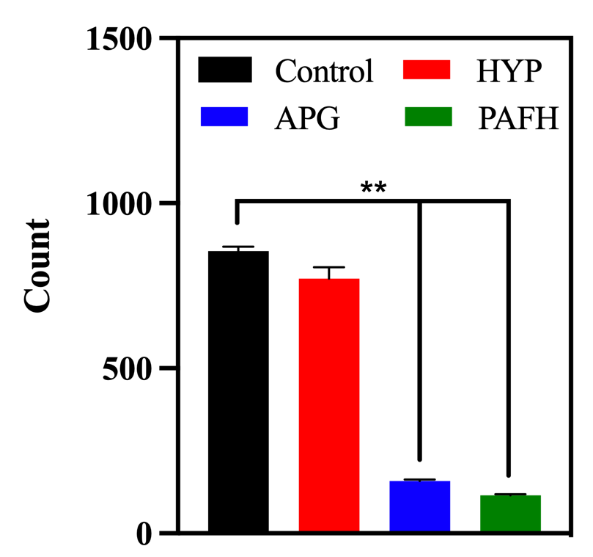


**Figure S7**. Quantitative analysis of Transwell cell migration after treatment with PAFH, APG and HYP with R+Y dual laser irradiation (mean ± SD, n = 3).


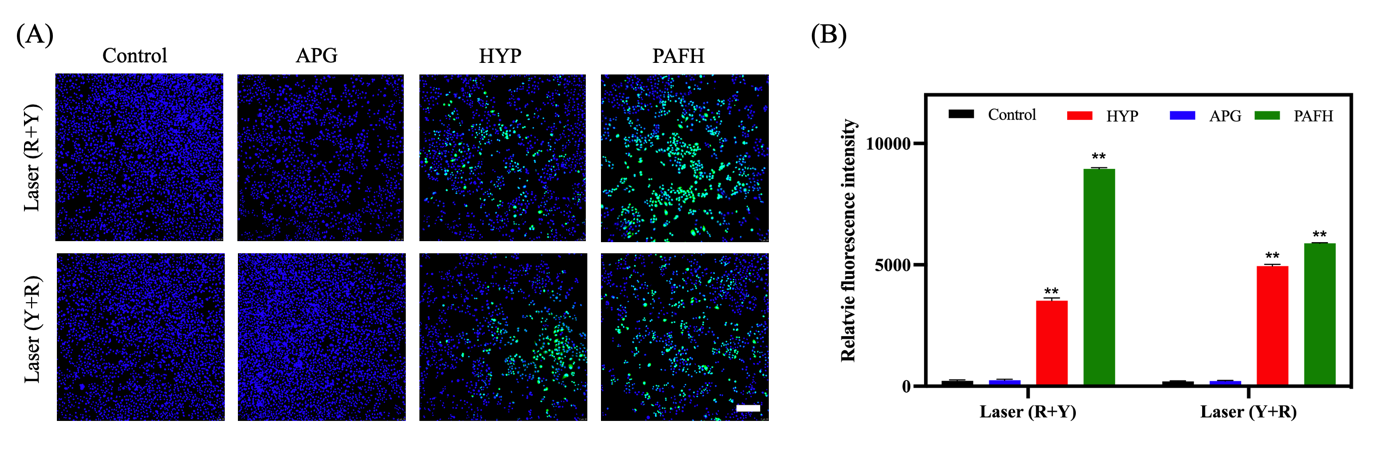


**Figure S8**. A) Fluorescence images and B) quantitative analysis of intracellular ROS induced by PAFH after different light irradiation sqeuence using DCFH-DA probe (mean ± SD, n = 3, scale bar: 200 μm).

**Figure S9**. MTT assay of cytotoxicity of different cellular uptake inhibitors (mean ± SD, n = 3).


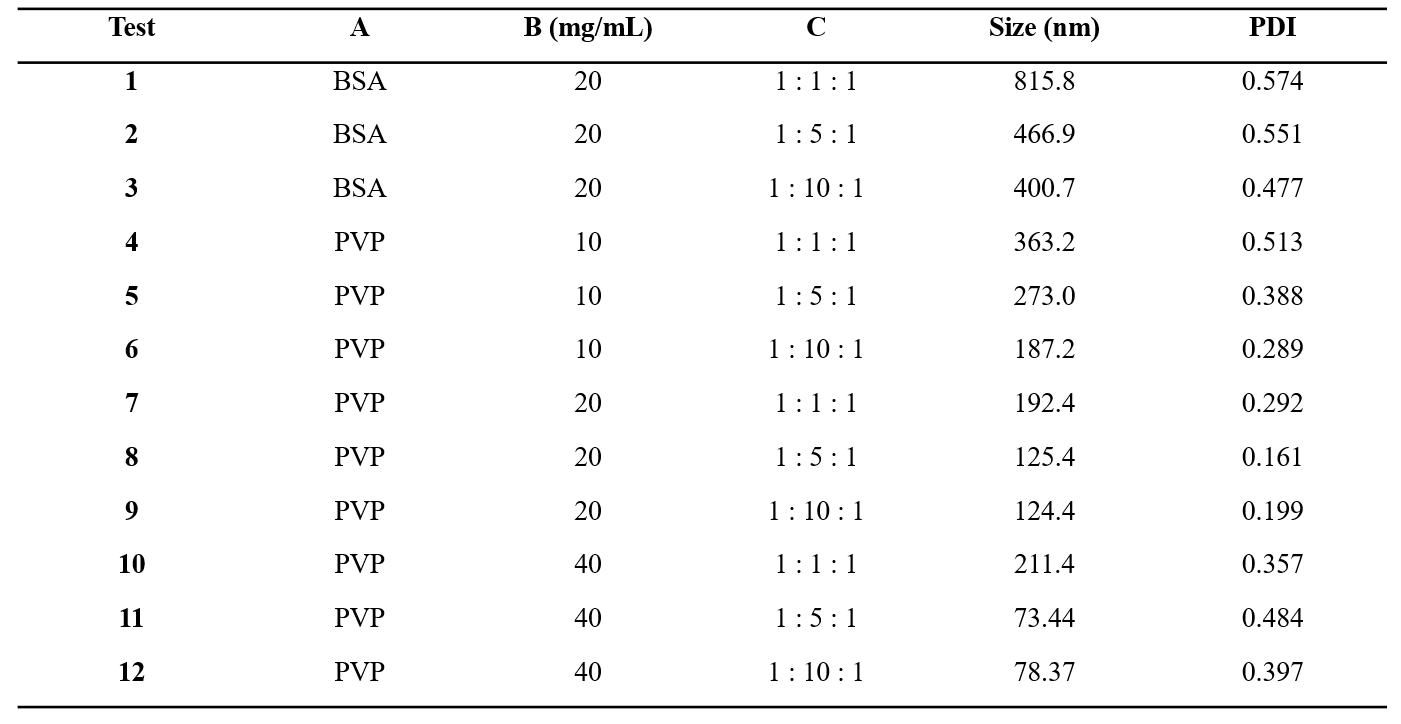


**Table S1**. Summarization of optimizing the particle size and PDI of PAFH. A: Types of dispersants. B: Concentration of dispersants. C: APG : Fe^3+^ : HYP ratios.
